# Efficacy and tolerability of four antimalarial combinations in the treatment of uncomplicated *Plasmodium falciparum *malaria in Senegal

**DOI:** 10.1186/1475-2875-6-80

**Published:** 2007-06-14

**Authors:** Babacar Faye, Jean-Louis Ndiaye, Daouda Ndiaye, Yemou Dieng, Oumar Faye, Oumar Gaye

**Affiliations:** 1 Laboratoire de Parasitologie-Mycologie, Faculté de Médecine, Pharmacie et Odontologie. Université Cheikh Anta Diop, Dakar, Senegal

## Abstract

**Background:**

In view of the high level of chloroquine resistance in many countries, WHO has recommended the use of combination therapy with artemisinin derivatives in the treatment of uncomplicated malaria due to *Plasmodium falciparum*. Four antimalarial drug combinations, artesunate plus amodiaquine (Arsucam^®^), artesunate plus mefloquine (Artequin^®^), artemether plus lumefantrine (Coartem^®^; four doses and six doses), and amodiaquine plus sulphadoxine-pyrimethamine, were studied in five health districts in Senegal.

**Methods:**

This is a descriptive, analytical, open, randomized study to evaluate the efficacy and tolerability of these four antimalarial combinations in the treatment of uncomplicated falciparum malaria using the 2002 WHO protocol.

**Results:**

All drug combinations demonstrated good efficacy. On day 28, all combinations resulted in an excellent clinical and parasitological response rate of 100% after correction for PCR results, except for the four-dose artemether-lumefantrine regimen (96.4%). Follow-up of approximately 10% of each treatment group on day 42 demonstrated an efficacy of 100%.

The combinations were well tolerated clinically and biologically. No unexpected side-effect was observed and all side-effects disappeared at the end of treatment. No serious side-effect requiring premature termination of treatment was observed.

**Conclusion:**

The four combinations are effective and well-tolerated.

## Background

Approximately 35% of in-patient and out-patient clinic attendance in Senegal are due to malaria [1]. Studies carried out by staff of the Parasitology Laboratory, Faculté de Médecine, Université Cheikh Anta Diop, Dakar, have demonstrated high levels of resistance to chloroquine, which was used as the first-line treatment of uncomplicated malaria and as chemoprophylaxis in pregnant women [2,3]. Clinical failure rates higher than 25% have been observed at more than half of the sites surveyed.

The development and rapid increase of chloroquine resistance in many countries led the World Health Organization to organize a technical consultation in April 2001[4], during which the association of two antimalarial drugs was recommended for the treatment of uncomplicated malaria, particularly those combining a classical antimalarial with an artemisinin derivative; such combinations are referred to as Artemisinin Combination Therapy (ACT).

In this study, the efficacy, safety and tolerability of three ACT for the treatment of malaria in Senegal were evaluated : artesunate plus amodiaquine (Arsucam^®^), artesunate plus mefloquine (Artequin^®^), and artemether plus lumefantrine (Coartem^®^; four doses and six doses). As a comparison, evaluation of combined therapy with amodiaquine plus sulphadoxine-pyrimethamine (SP) was also done; these latter antimalarial drugs are readily available in Senegal and were in use as a therapeutic strategy at the time of the study.

The objectives of the study were to evaluate the clinical and biological efficacy, safety and tolerability of these combinations in the treatment of uncomplicated malaria due to *Plasmodium falciparum *in Senegal, in order to generate data which could be used to determine the most effective antimalarial treatment policy.

## Methods

This descriptive, analytical, open, randomized study was carried out to evaluate the following antimalarial combinations in the treatment of *P. falciparum *uncomplicated malaria: artesunate-mefloquine(Artequin^®^; Laboratoire Mepha), artesunate-amodiaquine(Arsucam^®^; Laboratoire Sanofi Aventis), artemether-lumefantrine(Coartem^®^; Laboratoire Novartis), amodiaquine-SP (Pharmacie Nationale d'Approvisionnement du Sénégal).

The study was carried out during the transmission periods of 2002 and 2003 in five observation sites distributed according to the epidemiology of malaria transmission in Senegal: Richard Toll and Podor in the north, Guediawaye and Kaolack in the central zone and Velingara in the south. The transmission of malaria is moderate in these different zones with a peak during the rainy season.

### Study population

Patients were recruited from those presenting at the healthcare centers with clinical signs suggestive of malaria. After a thick peripheral blood smear for screening, patients of all ages who fulfilled the inclusion criteria laid down by WHO/2002 [5] were selected to take part in the study. The population size was determined according to the following criteria: the proportion of probable clinical failures with the antimalarial combinations studied should not be higher than 10%, for a level of confidence (P) of 95% and a precision (p) of 10%, taking into account patients who were excluded or lost to follow-up. Using these criteria, a minimum of 50 patients was required in each treatment arm at each site. The inclusion and exclusion criteria are described in the WHO/2002 protocol [5].

All patients gave their informed consent before participating in the study. The study protocol was approved by the ethics committee of the Ministry of Health

### Administration of antimalarial treatment

After inclusion, patients were weighed before being allocated to a treatment arm. All medication was administered under the direct supervision of a physician. If a patient vomited immediately after a dose, the same dose was re-administered within 30 minutes. Half of the dose was given if vomiting continued after the first 30 min. In the case of persistent vomiting, the patient was excluded from the study and sent to the medical team at the health center for management using the standard treatment in current use.

Dosages of the study drugs were as follows:

(1) artesunate-mefloquine: for adults, 200 mg/day artesunate plus 250 mg/day mefloquine, for 3 days; for children, 100 mg/day artesunate plus 125 mg/day mefloquine, for 3 days;

(2) artesunate-amodiaquine: 4 mg/kg/day artesunate plus 10 mg/kg/day amodiaquine, for 3 days. The dosages were also adjusted according to the age of the patient;

(3) artemether-lumefantrine: two dosages were administered: (i) in adults, four doses of four tablets repeated after 8, 24, 48 h; in children weighing 5–15 kg, one tablet repeated at 8, 24 and 48 h; (ii) six doses consisting of two daily doses for 3 days;

(4) amodiaquine-SP: 10 mg/kg/day amodiaquine for 3 days and half a tablet per 10 kg of SP as a single dose only on the first day.

All side-effects were monitored actively and passively during the study.

### Clinical evaluation and biological tests

Clinical evaluation and blood sampling from finger pricks for the preparation of thick peripheral blood smears were carried out on days 0, 1, 2, 7, 14, 21 and 28; 30% of the patients were also examined on day 42. Blood smears were stained with Giemsa and 200 leukocytes were read. Parasitaemia was expressed as the number of asexual parasites per microliter of blood according to the following formula: number of asexual parasites × 8,000/200.

Twenty-five percent of the patients were selected randomly and blood cell counts and levels of transaminases (Alanine amino transferase, ALAT, and Aspartate amino transferase, ASAT) and creatinine were determined on days 0, 14 and 28 to determine hepatic and renal function.

Biological tolerability was assessed by measuring haemoglobin (normal value: 12–17 g/dL), transaminases (ALAT between 5–30 UI/L, ASAT between 5–35 UI/L), and creatininaemia (normal value: 5–14 mg/L).

Blood collection on filter paper was carried out for all patients on days 0 and 28, and on all other days in cases of clinical or parasitological failure for genotyping the strain of *Plasmodium*. Genotyping was carried out in the Parasitology Laboratory, Faculté de Médecine, Université Cheikh Anta Diop, Dakar.

### Efficacy end-point criteria

The primary end-point criteria used to determine the efficacy of treatment were absence of parasites and clinical signs on days 14 and 28, and good clinical and biological tolerability. The secondary end-point criteria were the time taken for the parasites to disappear, and for gametocyte clearance.

### Definition of clinical and parasitological failure

The following definitions of clinical and parasitological failure were used:

#### Early therapeutic failure

This was defined as a patient who presented with danger signs between days 1 and 3, accompanied by parasitaemia; a patient with an axilliary temperature of > 37°C accompanied by parasitaemia on day 3; parasitaemia on day 3 > 25% that on day 0; and a patient with parasitaemia on day 2 higher than that on day 0.

#### Late therapeutic failure

This was defined as a patient presenting with danger signs between days 3 and 28, accompanied by parasitaemia; or a patient presenting with parasitaemia and a temperature > 37.5°C between days 3, 14 and 28.

#### Parasitological failure

Parasitological failure was defined as a patient presenting with parasites on day 28.

#### Overall therapeutic failure

This was defined as the sum of therapeutic failures plus parasitological failures.

#### Adequate clinical and parasitological response (ACPR)

ACPR was defined as the absence of parasites and clinical signs on day 28.

Patients who were considered to have failed clinically were given second-line therapy with quinine at a dose of 25 mg/kg/day for 5 days. All patients were subjected to clinical and biological tests during the 7 days following the administration of second-line therapy.

### Analysis of data

Data were collected and analysed using Epi info 6. Descriptive statistics were used to compare the demographic characteristics of the study population and their initial clinical and biological characteristics (temperature, geometric mean parasitaemia, etc.).

Clinical and biological data were compared with a confidence interval of 95%. All the clinical and laboratory data collected were subjected to quality control. The study was carried out according to standard operating procedures following the guidelines of good clinical practice.

## Results

### Study population and allocation of treatment

A total of 955 patients who fulfilled the inclusion criteria were included in the study. All patients gave their informed consent before participating in the study. Treatment was allocated as follows: 360 were given artesunate-amodiaquine, 145 were treated with artesunate-mefloquine, 140 were given four doses of artemether-lumefantrine, 149 were given six doses of artemether-lumefantrine, and 161 received amodiaquine-SP (Am-SP).

A comparison of the demographic and laboratory data at inclusion into the study showed that all treatments groups were practically identical (Table 1).

**Table 1:** Demographic and clinical characteristics of the study population according to treatment group on day 0.

	Artesunate-amodiaquine N = 360	Artesunate-mefloquine N = 145	Artemether-lumefantrine (four-doses) N = 140	Artemether-lumefantrine (six-doses) N = 149	Amodiaquine-sulphadoxine-pyrimethamine N = 161
Mean age (yrs)	15	15	12	13	12
Range	1–75	2–65	2–63	2–60	2–51
Mean wt (kg)	36	37	32	35	37
Range	5–102	11–110	12–83		6–89
Mean Parasitaemia (parasites/μL)	39800	27000	22000	15398	27000
Hb < 12 g/dL	68.6%	62.5%	96.7%	68.6%	70.3%
SGOT > 30 UI/L	15.2%	0%	9.7%	17.1%	0%
SGPT > 35 UI/L	0%	0%	0%	2.8%	0%
Creatininemia > 14 mg/L	0%	0%	0%	30%	0%

### Efficacy of treatment

On day 14, the ACPR was 100% for artesunate-amodiaquine, artesunate-mefloquine and Am-SP. For artemether-lumefantrine given as four doses, one case of clinical failure was observed resulting in an ACPR of 99.3% (Table 2). On day 14, the difference between this drug combination and the others was not statistically significant. On day 21, the percentage of clinical failures was 1.4% for artesunate-amodiaquine, 0.7% for artesunate-mefloquine, 3.6% for artemether-lumefantrine (four doses) and 0.6% for Am-SP. Once again, these differences between the groups were not statistically significant. On day 28, 16 cases of clinical failure and two cases of parasitological failure (88.8%) were observed in the artemether-lumefantrine (four-dose) group compared with 97.5%, 97.9% and 98.7% respectively for artesunate-amodiaquine, artesunate-mefloquine and Am-SP. At this timepoint, the difference between artemether-lumefantrine and the other treatment groups was statistically significant(p < 0.0001) (Table 3). There was no statistically significant difference when the other combinations were compared (artesunate-amodiaquine vs. artesunate-mefloquine; artesunate-amodiaquine vs. Am-SP; artesunate-mefloquine vs. Am-SP)(Table 3).

**Table 2:** Clinical and parasitological efficacy of the five antimalarial drug combinations

	**Artesunate-amodiaquine N = 360**	**Artesunate-mefloquine N = 145**	**Artemether-lumefantrine (four doses) N = 140**	**Artemether-lumefantrine (six doses) N = 149**	**Amodiaquine-sulphadoxine-pyrimethamine N = 161**
**Parasitological failure, day14**	0%	0%	0%	0%	0%
**Clinical failure, day14 (n)**	0%	0%	0.7% (1)	0%	0%
**ACPR, day 14**	100%	100%	99.3%	0%	100%
**Gametocyte carriage, day 14**	0%	0%	0%	3%	0%
**Parasitological failure, day 21**	0%	0%	0%	0%	0%
**Clinical failure, day 21 (n)**	1.4% (5)	0.7% (1)	3.6% (5)	0%	0.6% (1)
**ACPR, day 21**	98.6%	99.3%	96.4%	0%	99.4%
**Gametocyte carriage, day 21**	0%	0%	0%	0%	0%
**Parasitological failure, day 28 (n)**	0.8% (3)	0%	1.4% (2)	0%	0%
**Clinical failure, day 28 (n)**	0.3% (1)	1.4% (2)	11.4% (16)	0%	0.6% (1)
**ACPR, day 28**	97.5%	97.9%	82.8%	0%	98.7%
**Gametocyte carriage, day 28**	0%	0%	0%	0%	0%
**ACPR, day 28 after PCR**	100%	100%	96.4%	100%	100%
**ACPR, day 42 (30% of patients)**	100%	100%	100%	100%	100%

**Table 3:** Statistical comparison of the efficacy of the four different antimalarial drug combinations.

	Artesunate-amodiaquine	Artesunate-mefloquine	Artemether-lumefantrine (four doses)	Artemether-lumefantrine (six doses)	Amodiaquine-sulphadoxine-pyrimethamine
Overall efficacy	97.5%	97.9%	82.8%	100%	98.8%
Artesunate-amodiaquine	0	NS	P < 0.001	NS	NS
Artesunate-mefloquine		0	p < 0.001	NS	NS
Artemether-lumefantrine (four doses)			0	p < 0.001	p = 0.001
Artemether-lumefantrine (six doses)				0	NS
Amodiaquine-sulphadoxine-pyrimethamine					0

NS: not significant.

**Figure 1:**
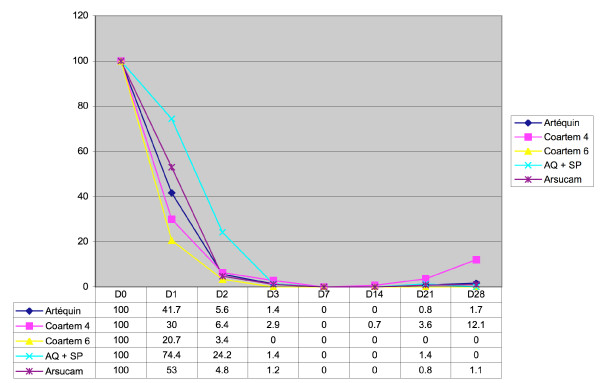
Parasitic clearance with the five antimalarial drug combinations.

Figure 1 shows that parasitic clearance on days 1 and 2 was slower with Am-SP compared with combinations containing an artemisinin derivative (p < 0.0001).

**Figure 2:**
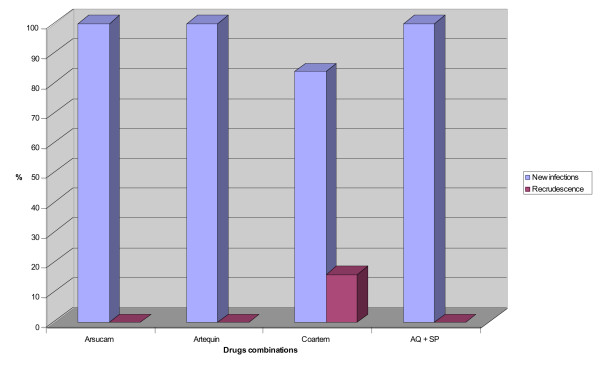
Genotyping of resistant strains isolated from patients treated with the different antimalarial drug combinations.

All the strains responsible for clinical or parasitological failure between days 21 and 28 were compared with the strains collected on day 0 by PCR using the marker MSP 1. Differences in strains were assessed on agarose gels using the specific markers MAD 20, K1 and RO 33. This comparison of strains with genetic markers before and after 'clinical failure' resulted in a correction of the results. No case of recurrence was observed after treatment with artesunate-amodiaquine, artesunate-mefloquine and Am-SP and 100% of clinical 'failures' were shown to be the result of re-infection. In contrast, the level of re-infection with artemether-lumefantrine (four-doses) was 84% (20/24) (Figure 2). Ninety-five percent of cases of clinical failure occurred in Kaolack. After correction for PCR results, a comparative analysis of the different combinations demonstrated that the difference between artemether-lumefantrine (four-doses) and the other combinations was no longer statistically significant (Table 4).

**Table 4:** Comparison of the efficacy of artemether-lumefantrine (four doses) with the other antimalarial drug combinations at day 28 after correction for PCR results

	Artesunate-mefloquine	Artesunate-amodiaquine	Artemether-lumefantrine (six doses)	Amodiaquine-sulphadoxine-pyrimethamine
Artemether-lumefantrine (four doses)	100%/96.4% p = 0.0019	100%/96.4% p = 0.065	100%/96.4% p = 0.0606	100%/96.4% p = 0.0492

When gametocytaemia was investigated, a reduction in gametocyte carriage was observed from day 3 for combinations including an artemisinin derivative, which acts against this parasitic stage. For Am-SP, however, an increase in number of patients carrying gametocytes was observed between days 0 and 7 (Table 5). From day 14, no gametocytes could be detected for any drug combination with the exception of artemether-lumefantrine (six-doses) (3%). By days 21 and 28, no patient had detectable gametocytes (Table 5).

**Table 5:** Evolution of gametocyte carriage according to antimalarial drug treatment through the study period

Day	0	3	7	14	21	28
Artesunate-amodiaquine	0%	0%	0%	0%	0%	0%
Artesunate-mefloquine	0%	2%	0%	0%	0%	0%
Artemether-lumefantrine (four doses)	2%	2%	0%	0%	0%	0%
Artemether-lumefantrine (six doses)	3%	6%	6%	3%	0%	0%
Amodiaquine-sulphadoxine-pyrimethamine	3%	10%	12%	0%	0%	0%

### Clinical and biological tolerance of treatment

The side-effects observed with each treatment combination were minor. These consisted mainly of mild gastralgia, dizziness, pruritus, asthenia and vomiting. These side-effects all disappeared at the end of treatment and did not require any specific therapy (Table 6). No serious side-effect requiring premature termination of treatment was observed.

**Table 6:** Biological tolerance to the five antimalarial drug combinations on days 14 and 28.

	Day	Artesunate-amodiaquine	Artesunate-mefloquine	Artemether-lumefantrine (four doses)	Artemether-lumefantrine (six doses)	Amodiaquine-sulphadoxine-pyrimethamine
Anemia (Hb < 12)	0	35/51	15/24	30/31	24/35	19/27
		68.6%	62.5%	96.7%	68.6%	70.3%
	14	40/51	17/24	20/31	24/35	21/27
		80.4%	70.8%	64.5%	68.6%	77.7%
SGOT > 30	0	9/59	0/27	3/31	6/35	0/27
		15.2%	0%	9.7%	17.1%	0%
	14	16/59	8/27	8/31	04/35	10/31
		27.1%	29.6%	25.9%	11.4%	32.2%
SGPT > 35	0	0/59	0/27	0/31	1/35	0/27
		0%	0%	0%	2.8%	0%
	14	3/58	3/27	4/31	1/35	2/31
		5.1%	11.1%	13%	2.8%	6.4%
Creatininemia > 14	0	0/59	0/27	0/37	30%	0/27
		0%	0%	0%		0%
	14	3/58	2/27	1/37	9/35	2/31
		5.1%	7.4%	2.8%	25.7%	6.4%

No severe alterations in renal or hepatic function were observed with any of the drug combinations under study. The transaminase increases observed on day 14 did not exceed 2.5 × normal values. Only two patients (one treated with artesunate-amodiaquine and one in the artemether-lumefantrine group) presented with a slight increase in creatinine levels without any serious clinical signs (Table 6).

## Discussion

The results of this study demonstrated that each of the five antimalarial drug regimens tested had good clinical and parasitological efficacy. When the efficacy results were corrected for PCR results, an ACPR of 100% was observed with artesunate-amodiaquine, artesunate-mefloquine, artemether-lumefantrine given as six doses, and Am-SP. Only artemether-lumefantrine given as four doses was associated with a failure rate of 3.6%. An initial study conducted in Senegal [6], did not detect any case of failure after 4 years revealing that four-dose artemether-lumefantrine seems to have progressively lost its efficacy. It should be noted, however, that most cases of failure were observed around Kaolack, which is an important crossing point between adjacent countries (The Gambia, Guinea and Mali.), with a well established practice of self-medication and a readily accessible alternative drug market. The highest level of failure due to chloroquine in Senegal has also been noted in this area.

In Asia, studies have demonstrated that in areas where transmission is high and where the population has a high level of immunity to malaria, a therapeutic approach based on four doses of artemether-lumefantrine gave good results. Nevertheless, in non-immune populations (especially in south-east Asia) this therapeutic approach was associated with a failure rate of approximately 20%. This failure rate was reduced to less than 5% by using a dosage schedule based on six doses [7]. The failure rate with artemether-lumefantrine given as four doses [8,10] forced us to change to a six-dose regimen which gave a better efficacy in this study.

The excellent efficacy observed with artesunate-mefloquine at the present dosage, seems to demonstrate good activity against the asexual form of the parasite. Nevertheless, under the endemic conditions in our region, transmission levels also confer a degree of protection which can act synergistically with a drug. No case of re-infection was observed after a follow-up period of 42 days. It appears, therefore, that the strains of *P. falciparum *encountered in this study were highly susceptible to mefloquine despite the small dose used (15 mg/kg mefloquine). However, to prevent a reduction in parasite susceptibility, an increase in the dosage of mefloquine to 25 mg/kg was necessary, as shown previously in other studies [9].

The combination of Am-SP is still effective in Senegal and in central Africa [11], while in east Africa cases of resistance have been demonstrated [1,3,12,14].

Since 2003, the Programme National de Lutte contre le Paludisme du Sénégal (National Malaria Control Programme) has used the Am-SP combination instead of chloroquine as a temporary strategy in the treatment of uncomplicated malaria. However, it is believed that the widespread use of this combination in addition to the use of SP alone in pregnant women together with population movements is likely to lead to the appearance of therapeutic resistance to these drugs. Indeed, a study carried out in Senegal on molecular markers of resistance to SP has demonstrated the presence of triple mutations for dhfr and dhps [15].

The gametocidal effect of artemisinin derivatives against gametocytes has been demonstrated in other studies [14] and has been confirmed in the current study; no gametocytaemia was detectable in patients treated with an artemisinin derivative from day 3. Only the combination of Am-SP resulted in increased levels of gametocytes until day 7.

Genotyping of resistant stains using MSP1 and the alleles K1, RO 33 and MAD 20 enabled us to differentiate cases of recurrence from new infections.

All drug combinations were well tolerated in this study. Only minor adverse events were observed, as seen in other studies [16,17] and these did not require premature termination of treatment or specific medical care. All adverse events disappeared at the end of treatment and no serious adverse event was observed.

Similarly, clinical and biological tolerability was good for all drug combinations. No unexpected adverse events were encountered and all disappeared at the end of treatment. No serious adverse event requiring premature termination of treatment was observed. However, it is important to carry out further pharmacovigilance studies before these combinations are used more widely.

Artemisinin derivatives in combination with classical antimalarial drugs, which are still effective as monotherapy, represent, therefore, the best options for the treatment of malaria in situations where chloroquine resistance exists. Possible hurdles to their use as treatment strategies for uncomplicated malaria include their relatively high cost and their availability in the context of a high worldwide demand. Considerable commitment by the pharmaceutical industry and increased participation of international organizations are necessary to give the population of these poor countries access to antimalarial drug combinations based on artemisinin derivatives.

On the basis of these results, Senegal has modified its approach to treatment of malaria since 2004 and has adopted artesunate in combination with amodiaquine as first-line therapy for the management of uncomplicated malaria. However, a regular monitoring of this strategy is essential because therapeutic failures were documented in the south of Senegal [18].

## Authors' contributions

FB : Co principal investigator. Supervised the study, the data's coll, made molecular biology study and wrote the paper

NJL: Co investigator, Statistical analyser

ND: Molecular Biology advisor. Participate in the molecular biology study

DY: Co investigator

FO: Co investigator

GO: Co principal investigator. Initiated the study, revised the paper

All the authors have read and approved the last manuscript
